# Enhancing Workplace Wellness Efforts to Reduce Obesity: A Qualitative Study of Low-Wage Workers in St Louis, Missouri, 2013–2014

**DOI:** 10.5888/pcd12.140405

**Published:** 2015-05-07

**Authors:** Jaime R. Strickland, Amy A. Eyler, Jason Q. Purnell, Anna M. Kinghorn, Cynthia Herrick, Bradley A. Evanoff

**Affiliations:** Author affiliations: Amy A. Eyler, Jason Q. Purnell, George Warren Brown School of Social Work, Washington University, St. Louis, Missouri; Anna M. Kinghorn, Cynthia Herrick, Brad A. Evanoff, Washington University School of Medicine, St. Louis, Missouri.

## Abstract

**Introduction:**

The objective of this study was to examine workplace determinants of obesity and participation in employer-sponsored wellness programs among low-wage workers.

**Methods:**

We conducted key informant interviews and focus groups with 2 partner organizations: a health care employer and a union representing retail workers. Interviews and focus groups discussed worksite factors that support or constrain healthy eating and physical activity and barriers that reduce participation in workplace wellness programs. Focus group discussions were transcribed and coded to identify main themes related to healthy eating, physical activity, and workplace factors that affect health.

**Results:**

Although the union informants recognized the need for workplace wellness programs, very few programs were offered because informants did not know how to reach their widespread and diverse membership. Informants from the health care organization described various programs available to employees but noted several barriers to effective implementation. Workers discussed how their job characteristics contributed to their weight; irregular schedules, shift work, short breaks, physical job demands, and food options at work were among the most commonly discussed contributors to poor eating and exercise behaviors. Workers also described several general factors such as motivation, time, money, and conflicting responsibilities.

**Conclusion:**

The workplace offers unique opportunities for obesity interventions that go beyond traditional approaches. Our results suggest that modifying the physical and social work environment by using participatory or integrated health and safety approaches may improve eating and physical activity behaviors. However, more research is needed about the methods best suited to the needs of low-wage workers.

## Introduction

Obesity, a major risk factor for diabetes, affects more than one-third of adults in the United States and is associated with several demographic and socioeconomic factors, including low income (1). Several studies have found that obesity rates are generally higher among working class occupations than professional occupations, even after controlling for demographic factors (2,3).

From a sociological perspective, the environments in which people live and work are strong influences on obesity and diabetes (4,5). The work environment is especially important because many adults spend a significant amount of time at work and because obesity affects employers through reduced productivity and absenteeism as well as increased health care costs and disability (6). Numerous studies acknowledge the negative health consequences of workplace factors such as stress, low autonomy, poor coworker and managerial support, and unhealthy physical work environments (2,7). These workplace risk factors may be more common in low-wage and working-class jobs and may explain some occupational differences in obesity prevalence (2,8).

Promoting health through worksite wellness programs is a national priority. The Affordable Care Act creates new incentives to promote employer wellness programs and encourage opportunities to support healthier workplaces (9). The National Institutes of Health and the Centers for Disease Control and Prevention have targeted worksites as a priority location for health interventions because they offer an efficient means of delivering and evaluating programs and provide opportunities to reach socially disadvantaged populations (10,11). However, data for the effectiveness of workplace health programs are limited and may not be generalizable to all types of workers (6,11–13). National data show that blue-collar and service workers are less likely to work for an employer who offers health promotion activities and are less likely to participate in such programs when offered (14).

This study focused on a little-studied health disparity — workplace health promotion among low-wage workers. The objective of the study was to examine through interviews and focus groups 1) worksite culture, environment, and policies that influence healthy eating and physical activity; and 2) barriers that reduce worker participation in workplace health promotion programs. An understanding of how the workplace affects health behaviors is can inform design of effective interventions to reduce and prevent obesity.

## Methods

We partnered with a large health care system and a national labor union representing retail workers to recruit study participants. Qualitative data collection included interviews with key informants (eg, employer representatives, union leaders, benefits administrators) and worker focus groups with both partner organizations. The workforce in the union was relatively homogenous with regard to income and included workers in jobs such as cashier and merchandise stocker. Within the health care system, we targeted hospital work departments and locations that employed a large proportion of low-wage workers, including housekeepers, patient care technicians, and food service workers. This study was approved by the Washington University institutional review board.

We interviewed 10 individuals from the union partner: 4 local union leaders, 5 store representatives, and 1 health benefits administrator. Key informants were recruited in person or through email, and interviews were conducted in person or over the telephone. We asked about current and previous wellness initiatives offered to employees, employee participation in these initiatives, and potential barriers to participation. Informants were also asked about workplace factors that influenced health behaviors (ie, physical activity and healthy eating) and employee attitudes about health and wellness.

We conducted a total of 9 focus groups involving 61 workers. Twenty hospital employees (4 men and 16 women) participated in 4 groups. Forty-one unionized retail workers including 12 men and 29 women participated in 5 focus groups. Focus group participants were recruited through their work department, store, or local union hall. The research team attended union meetings to recruit members in person and posted flyers in break rooms at selected stores and hospital departments. We used a semistructured script to guide focus group discussions. The scripts covered 11 broad domains with follow-up questions and prompts for each domain (Table 1). All group discussions were audio recorded and transcribed. Transcriptions were entered into QSR International’s NVivo 10 software (QSR International Pty Ltd), and all were coded by 2 independent raters using a predefined code book based on the domains in the focus group script. After initial coding and consensus of all transcripts, we applied a phenomenological approach for data analysis to find the “essence” or common themes across individual experiences (15). The purpose of the thematic analysis was to answer 2 questions: “what impacts healthy eating and physical activity” and “what can be modified at the workplace?” Through systematic review and discussion, codes were merged and grouped under main themes. Each transcript was re-read and re-coded for consistency. 

**Table 1:** Focus Group Domains and Questions, Qualitative Study of Low-Wage Workers, St. Louis, Missouri, 2013–2014

Domain	Questions	Examples, Clarification, Follow-ups, Probes
Work schedule	Tell us about a typical work day.	How many hours do you usually work? What opportunities do you have for breaks?
Healthy eating priority	Is eating healthy a personal priority for you?	Do you try to eat healthy? What do you do at home to eat healthy? Are you satisfied with your diet?
Eating at work	When do you eat while at work? What do you eat while at work?	How do you decide what you will eat while at work?
Exercise priority	Is regular exercise a personal priority for you?	Do you try to exercise? How often, where do you exercise? Are you satisfied with your level of physical activity?
Physical activity at work	What kind of physical activity/exercise do you do at work?	Do you do anything in addition to your normal work routine to be more physically active? (eg, take the stairs, walk during break times)
Worksite health facilitators	What aspects of work at [organization] seem to help you or your coworkers stay healthy while at work?	Current wellness or safety programs that are helpful? Helpful aspects about physical environment or company policies that promote health? What qualities of your job make you feel good? Keep you fit? Do your work relationships contribute to health? How?
Worksite health barriers	Which aspects of your work or work environment get in the way of being healthy?	Are there things about your work tasks or the way work is organized that make it difficult for you to take care of your health? What aspects of work prevent you from engaging in healthy activities outside of work?
Health concerns	What health issues are you most concerned about for yourself?	How concerned are you about missing work due to illness/injury?
Current wellness programs	Are you aware of any health and wellness programs currently or previously offered to employees? (ie, weight-loss, smoking cessation)	Have you or any of your coworkers participated in any of these wellness programs?
Communication	How does your employer communicate important information to you?	What about health information?
Future workplace programs	How likely are you to participate in workplace wellness programs in the future? What about nutrition and exercise programs, specifically?	What factors might influence your decision to participate? (ie, cost, location, other). How can your employer/union do a better job of promoting wellness in employees?

## Results

### Key informant interviews

The informants indicated that very few wellness programs related to weight management were offered to retail workers. The union-sponsored health plan covered some costs for nutritional counseling, but that benefit was not well advertised. The employer-sponsored initiatives such as an onsite gym or weight loss programs were primarily available to employees in the corporate offices, not to workers in retail stores. Both the union and employer representatives recognized the need for workplace wellness programs but were unsure about how to proceed with developing and implementing a program to reach their diverse and widespread workforce.

Informants described various programs available to employees but noted several barriers to effective program implementation, including lack of management commitment at some levels, limited budgets, and communication and advertising limitations. One informant described results of a focus group conducted among employees of 1 hospital department regarding awareness of existing wellness programs and preferred methods of communication; results indicated that most workers were unaware of the wellness program and did not regularly use company email, which was the primary method of communicating information about the wellness program. Workers preferred to get information via personal email, text message, or in person. Workplace wellness efforts within the health care organization varied by worksite; some sites were more successful in promoting and delivering their wellness initiatives than others. Informants thought the size of organization and motivation of appointed representatives for each location influenced program success. An informant from a smaller hospital mentioned several successful wellness initiatives at her location, including an onsite gym, exercise classes, and 2 weight-loss challenges each year, and an informant from a larger hospital discussed struggles to find effective communication methods to reach all worker groups.

### Worker focus groups

The final list of themes from the focus group analysis included 10 work-related themes and 10 general themes (Table 2). Workers commonly discussed how their job characteristics contributed to their health. For example, they mentioned that physical demands and stress of their jobs left them too exhausted or unmotivated to exercise or plan healthy meals (Table 3). Many also described how the physical environment affected their health (eg, small work area, concrete floors). Past or current company programs and priorities was another common theme identified, although details varied by group. Overall, the retail workers talked about lack of wellness programs; some mentioned store weight-loss competitions and previous company campaigns but felt that their employers and union did not prioritize health and wellness. Responses of the health care worker groups differed; those working in a large hospital setting were much less aware of wellness initiatives and felt less company or management support for health promotion. Many were aware of the onsite gym and the weight-loss program, but cost, work schedule, and home responsibilities made it difficult to participate. Conversely, a group working in a smaller clinic felt tremendous upper-management support and described numerous workplace supports, including a produce garden at the worksite, access to exercise equipment, afternoon stretch breaks, and healthy potluck lunches.

**Table 2:** Main Focus Group Themes and Number of Associated Coded References, Qualitative Study of Low-Wage Workers, St. Louis, Missouri, 2013–2014

Theme (N)^a^	Topics Included
**Work-related theme**	
Job characteristics (196)	Physical and mental demands, stress, physical environment, safety, workplace rules
Company priorities and programs (165)	Company health promotion programs, perception of company priorities for employee health
Food options (105)	Food options at work (free or available for purchase)
Communication (92)	Communication of health information, preferred methods of communication
Work schedule (75)	Schedule, time of day worked
Social support/accountability (72)	Desire for social support or being held accountable, camaraderie
Management support (48)	Perception of management support, employee–management relationships
Facilities (45)	Aspects of current facility related to health or suggestions for changes to facilities
Breaks (40)	Relationship between breaks and health behaviors
Other (24)	Knowledge from job, suggestions for general workplace changes
**General theme**	
Intrapersonal (168)	Motivation, willpower, impulse, desire to be healthy/look good
Financial (132)	Company discounts, cost of food, gym memberships
Home life (94)	Cooking at home, food restrictions, outside environment, other priorities/responsibilities
Time (75)	Not enough time, availability of quick options
Energy (53)	Lack of energy, need energy
Food preferences (49)	How eating habits/preferences affect food choices
Planning (45)	Lack of routine, difficulties of planning, reasons behind planning or not planning
Convenience (33)	Convenience of food options, wellness programs; choices that require little effort
Personal health (20)	Physical and mental health as barriers to eating well or participating in physical activity
Transportation (16)	Influence of transportation on participation in wellness programs

a N = number of times this theme was referenced.

**Table 3:** Sample Comments and Coded Themes, Qualitative Study of Low-Wage Workers, St. Louis, Missouri, 2013–2014

Comment	Theme Coded
“If any employer is really serious about wanting a healthier work environment and employees then they have to make sure they have the proper rest time. I am squishing my two 15-minute breaks together to make my half-hour lunch.”	Company priorities and programs, breaks
“I think I would [go to the workplace gym] because I think somebody would go with me from here. You’d have a buddy. You have so many friends inside of [the store]. I mean I have friends at other [stores] and I could be like ‘Hey, meet me at our gym.’”	Social support-accountability, company priorities and programs
“When I first started working here I thought it was the oddest thing that I would walk to the cafeteria and I would see nurses, techs, eating when they are walking, eating at the elevator . . . but now I know why they do that, you know, ‘cause sometimes that is all the time they get.”	Breaks, time, job characteristics
“And that's another thing, they got a lot of good different varieties during the day, but at night, there is not much to choose from.”	Work schedule, food options
“But it is funny because they put [smoking cessation ads] in the break room but the smokers don’t go in the break room, they go outside. So nobody saw it.”	Communication
“And I have to say, she [upper-level manager] don’t throw it down your throat . . . I don’t think anybody does. They put the option out there and it’s your choice to participate or not. They give us the resources to use and they say here, now it is up to you They will promote something [monthly] that most of us probably didn’t know . . . to help us.”	Company priorities and programs, management support
“I feel like not having set schedules makes it kinda hard to exercise, because sometimes you work early in the morning, sometimes you’ll work late at night. Throws off your sleep schedule.”	Work schedule
“If you’re too tired and you’re stressed out, you don’t want to do anything but eat that fattening food and curl up in a little ball and go to bed. You don’t plan for tomorrow; you just have to get through the day.”	Planning, energy
“I’m a food addict, I’ll admit it; I like food. I have all intents and purposes of going to the salad bar and picking the good lettuce, the good stuff, the good fruits, the good vegetables, but man as soon as that [BBQ smoker] hits me, I’m gone!”	Intrapersonal, food preferences
“I prepare my lunch every morning. I work and then I actually walk every day . . . up to 5, 6, 7 miles every day . . . except for today because all of us had double shifts. So that's it, I have the will power, I’m not gonna lie. Most people don’t know me, but I’ve dropped a ton of weight. I was quite large and I just made a goal this year that I was gonna take care of myself.”	Intrapersonal, planning, work schedule

Workers also discussed schedules and breaks as having a significant impact on their healthy eating and physical activity. For many retail workers, their schedules varied week-to-week, making it difficult to maintain any routine. Workers from both organizations stated that short and interrupted breaks made it difficult to eat healthy. They discussed how food options —healthy or unhealthy and purchased or provided for free (eg, incentive lunches, holiday parties) — affected their eating behaviors at work. Workers from both organizations felt that their workplaces had a lack of quick, convenient, and low-cost healthy food options. Moreover, in all groups we heard that free food was almost always unhealthy. Nearly all workers commented that social support and accountability to coworkers would improve their ability to initiate and maintain healthy behaviors.

General themes were those that may be related to the workplace but also extended into workers’ personal lives. For example, workers often discussed how intrapersonal factors (eg, motivation, willpower) and home life (eg, responsibilities, family support) affected their health behaviors both in the workplace and at home. Workers often discussed how their jobs influenced their health in terms of not having the money, time, or energy to exercise or plan healthy meals. Some workers also discussed the roles that health issues and transportation played in initiating and sustaining healthy behaviors.

## Discussion

This study highlights factors related to obesity as described by 2 low-wage work groups; our findings are consistent with results from a similar study among low-wage workers in various industries (8). The workplace was often viewed as a barrier to healthy eating and physical activity; however, workers supported the concept of workplace health promotion and offered suggestions for overcoming many of the identified barriers. As demonstrated in this study, the workplace may be effective in engaging populations at risk for obesity and related illnesses, though it may be necessary to go beyond traditional workplace wellness approaches. Using more innovative methods may increase program reach, effectiveness, and sustainability.

Policy changes have increasingly been recognized as essential components of worksite health promotion (16) and are more sustainable than individual-level behavior interventions (17). Policies promoting a culture and environment conducive to reducing obesity can be a strong catalyst to behavior change. These can include top-level policies, such as offering a health care plan that has wellness options or implementing organizational policies that provide for access to low-cost healthy foods at the worksite, encourage active transportation to and from work, or allow for flexible work schedules to encourage lunch or break-time physical activity. The work environment (both indoor and outdoor) is also an important component of behavior change and can have a significant impact on behavior choice (18). An environment that encourages less sedentary work and more physical activity could include well-placed and maintained stairwells for stair use versus elevators or distant parking.

Changes solely in the workplace environment may not be enough to encourage healthy behaviors (19). Health behavior decisions are affected by the social context in which they are made, such that the social support and social norms surrounding a health issue have a substantial effect on how that health behavior is perceived. Changing social norms and fostering a supportive work environment for the desired behavior is a necessary complement to the other levels of intervention. Social norms have been studied as a way to promote nutrition (20) and physical activity (21).

Workplace participatory approaches may foster social support and help to overcome organizational and employee barriers to program success. Most worksite weight-loss programs have relied on a top-down approach, rather than a participatory approach based on employee involvement in the design of interventions (22). In workplaces where employees generally have little influence on their work environment, similar to those sampled in this study, participatory approaches can result in better program implementation and subsequent health improvement (22). The recently described Healthy Workplace Participatory Program (HWPP) includes work environment changes, as well as healthy eating and physical activity interventions (23). A small study based on HWPP found promising changes in behaviors and weight loss in a pre–post evaluation of a participatory worksite intervention (24). To our knowledge, this HWPP-based study is the only controlled study to date using a worker health participatory program to attain weight loss. Future research should implement and evaluate workplace participatory interventions for weight loss.

Workplace wellness programs should also use effective communication strategies to engage workers from diverse work groups and backgrounds. As demonstrated with the health care system in this study, many low-wage workers were not aware of the wellness programs that were available to them. The same programs, however, have good participation from other work groups in the health care organization, primarily because of the method of communication. Rapid changes in information technology have enabled new interventions that use mobile telephones and other mobile devices (mHealth). These techniques show great promise for weight reduction in low-income populations (25), and such interventions are readily scalable to larger populations (13).

Although we did not directly ask about incentives, several participants discussed monetary incentives as a possible motivator to eat healthy and exercise. The use of incentives is common in workplace wellness programs; employers could maximize the benefits of incentives by incorporating lessons from behavioral economics. For example, the increasingly popular approach of delivering incentives through health insurance premium adjustments is unlikely to be as effective as more frequent and immediate rewards for behavior. This is because people tend to discount the future, meaning that they respond more readily to immediate than delayed costs and benefits (26). The participants in our study commonly discussed cost as a barrier to eating healthy and exercising. As suggested by others (27), low-income workers may be more likely to change and sustain healthy behaviors if provided with financial support for healthy food and participation in other weight-loss activities. Employers should also be aware of the limitations of incentives for behavior change. Recent reviews have shown behavioral effects to be relatively short-lived after incentives are removed (27), and considerable attrition is found in workplace programs for weight loss (28). More research is needed to determine the optimal timing, magnitude, and structure of incentives, but results to date suggest that incentives may need to be an ongoing feature of the workplace to have maximum impact.

Finally, employers may consider integrating traditional occupational safety and health programs (ie, those that focus on health hazards unique to the workplace) with health promotion and wellness programs (ie, those that focus exclusively on lifestyle factors off the job). The Total Worker Health program was launched by the National Institute of Occupational Safety and Health (NIOSH) to support the development and adoption of research and best practices to integrate these approaches and address health and safety risks at multiple levels, including the work environment (physical and organizational) and individual behaviors. This integrative approach may lead to greater adoption of interventions by management and workers and hence to improvements in the health of workers (11), but more research is needed to evaluate both the development process and the effectiveness of integrated programs (29).

The results of this study can help inform future worksite interventions for low-wage workers; however, our study has several limitations. First, we collected data from key informants who could be contacted or agreed to be interviewed. Second, although the participants in the focus groups represented a range of positions and worker groups, they were limited to those available during the implementation of the focus group discussions. Although using a convenience sample may be a limitation, those who elected to participate in the interviews or focus groups were able to provide helpful insights on the topic. Future intervention planning would need to be preceded by additional input from a broader participant base. Third, the information we collected may not be generalizable to other health conditions or work settings. Despite these limitations, the key informants and focus group participants provided rich and potentially actionable information on addressing obesity at the worksites of these worker populations.

Workplaces can provide an effective venue for engaging low-income populations at risk for obesity and related illnesses. Results of this study suggest that future worksite interventions for low-wage workers can improve reach, effectiveness, and sustainability if they embrace more innovative methods than those used in current workplace wellness programs. Future interventions should address workplace policies and environment and social norms that affect health behavior decisions. Communication strategies and financial incentives should be better aligned with the needs of low-wage workers. Workplace participatory programs are a promising approach to engage workers in health improvement.
